# Normal Tissue Injury Induced by Photon and Proton Therapies: Gaps and Opportunities

**DOI:** 10.1016/j.ijrobp.2021.02.043

**Published:** 2021-02-25

**Authors:** Pataje G. Prasanna, Kamila Rawojc, Chandan Guha, Jeffrey C. Buchsbaum, Justyna U. Miszczyk, C. Norman Coleman

**Affiliations:** * Radiation Research Program, Division of Cancer Treatment and Diagnosis, National Cancer Institute, Bethesda, Maryland; † The University Hospital in Krakow, Department of Endocrinology, Nuclear Medicine Unit, Krakow, Poland; ‡ Department of Radiation Oncology, Albert Einstein College of Medicine and Montefiore Medical Center, Bronx, New York; § Department of Experimental Physics of Complex Systems, Institute of Nuclear Physics, Polish Academy of Sciences, Krakow, Poland.

## Abstract

Despite technological advances in radiation therapy (RT) and cancer treatment, patients still experience adverse effects. Proton therapy (PT) has emerged as a valuable RT modality that can improve treatment outcomes. Normal tissue injury is an important determinant of the outcome; therefore, for this review, we analyzed 2 databases: (1) clinical trials registered with ClinicalTrials.gov and (2) the literature on PT in PubMed, which shows a steady increase in the number of publications. Most studies in PT registered with ClinicalTrials.gov with results available are nonrandomized early phase studies with a relatively small number of patients enrolled. From the larger database of nonrandomized trials, we listed adverse events in specific organs/sites among patients with cancer who are treated with photons and protons to identify critical issues. The present data demonstrate dosimetric advantages of PT with favorable toxicity profiles and form the basis for comparative randomized prospective trials. A comparative analysis of 3 recently completed randomized trials for normal tissue toxicities suggests that for early stage non-small cell lung cancer, no meaningful comparison could be made between stereotactic body RT and stereotactic body PT due to low accrual (NCT01511081). In addition, for locally advanced non-small cell lung cancer, a comparison of intensity modulated RTwith passive scattering PT (now largely replaced by spot-scanned intensity modulated PT), PT did not provide any benefit in normal tissue toxicity or locoregional failure over photon therapy. Finally, for locally advanced esophageal cancer, proton beam therapy provided a lower total toxicity burden but did not improve progression-free survival and quality of life (NCT01512589). The purpose of this review is to inform the limitations of current trials looking at protons and photons, considering that advances in technology, physics, and biology are a continuum, and to advocate for future trials geared toward accurate precision RT that need to be viewed as an iterative process in a defined path toward delivering optimal radiation treatment. A foundational understanding of the radiobiologic differences between protons and photons in tumor and normal tissue responses is fundamental to, and necessary for, determining the suitability of a given type of biologically optimized RT to a patient or cohort.

## Introduction

Radiation therapy (RT) is used to treat about half of all patients with cancer, often in combination with chemotherapy, immunotherapy, and/or surgery.^1^ RT use is projected to remain at least at the same level in the coming decades, with its accessibility for patients with cancer expected to increase among low- to middle-income countries.^2^ Most new treatment facilities or upgrades to existing facilities will be to linear accelerators. There are also significant efforts to establish proton therapy (PT) facilities and a few carbon ion treatment centers, mostly in high-income countries. According to the Particle Therapy Cooperative Group, as of December 2020, there are 110 particle therapy facilities in operation worldwide, 37 under construction, and 28 in various stages of planning. PT has emerged as a treatment option for certain types of cancers.^3–6^

Despite advances in radiation oncology, patients still experience adverse events (AEs). Some examples of therapy-related AEs have been previously reviewed.^7–10^ With a steady increase in population growth, cancer occurrence, improvements in screening, detection, treatment, and outreach, cancer survivors are a growing population.^11^ As survivors live longer, late effects (eg, cognitive impairment, hearing loss, tissue fibrosis, and endocrinopathy) may manifest, as well as secondary cancers.^12^ Furthermore, recent comparative risk analyses of a second cancer diagnosis after primary cancer treatment among pediatric and adult patients indicated that the risk of second cancers was similar after intensity modulated RT (IMRT) and 3-dimensional conformal RT, whereas proton beam RT was associated with a lower risk of second cancers.^13^ There is an unmet need to recognize the possible differences in the mechanisms of radiation injury and normal tissue toxicities with different radiation types.^14^

For this review, we obtained data from 2 publicly available databases: U.S. National Library of Medicine’s ClinicalTrials.gov (https://clinicaltrials.gov) for the analysis of clinical trials and PubMed using iCite (https://iCite.od.nih.gov/; Office of Portfolio Analysis, NIH, Bethesda, MD), a web application to access bibliometric information for publications.^15^ We highlight the major issues with AEs and normal tissue toxicities with a focus on PT trials and identify important gaps. Further outlining the differences in the mechanisms of cellular injury and biological damage between PT and photon therapy, we advocate consideration of biological variables alongside physical parameters as determinants of normal tissue toxicities and exploit these differences to achieve accurate precision radiation medicine.^16^ This review assesses the published literature on PT and clinical trials that compared proton versus photon therapy on normal tissue toxicities. This analysis will help in better designing future trials as the importance of normal tissue toxicities and the need for personalization of RT are increasingly recognized.

### Clinical Studies

**Fig. 1.**
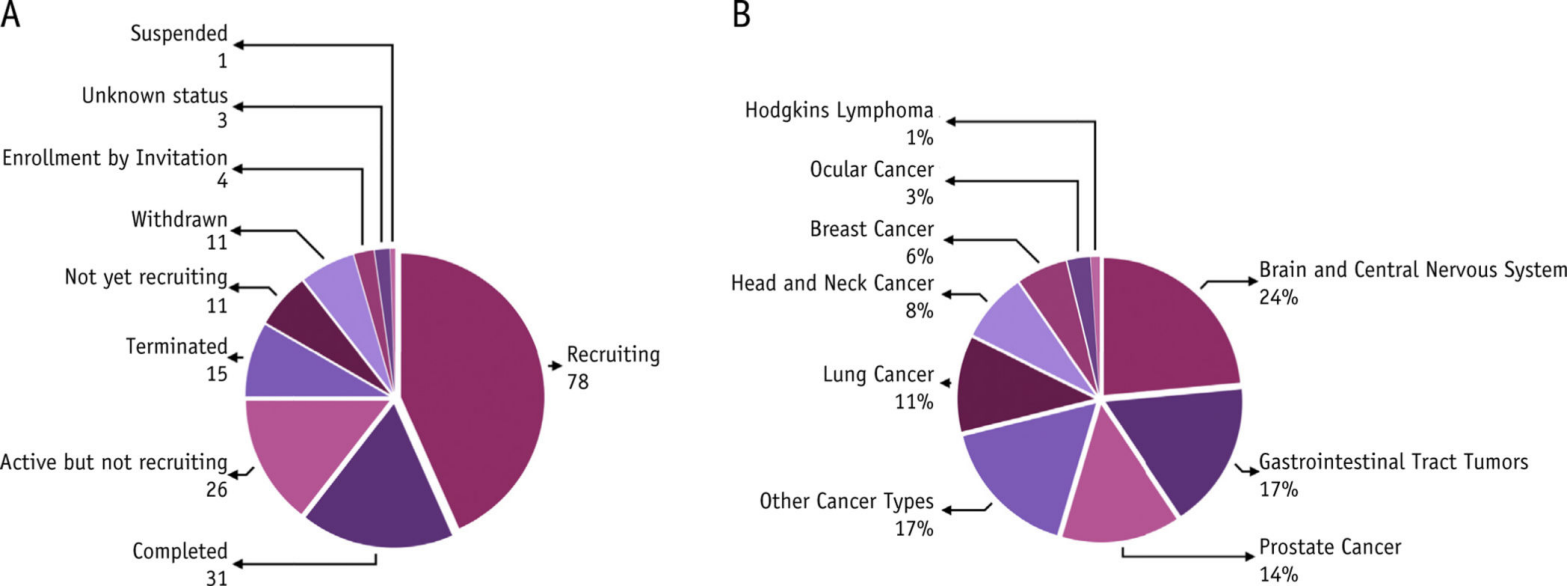
Details of clinical trials conducted with proton therapy as registered with clinicaltrials.gov. (A) Clinical trials conducted with proton therapy, their overall status, and the number of trials in a specific stage. (B) Percentage of trials for different organ/sites.

A head-to-head comparison of AEs from PT with photon therapy is desired but difficult due to differences in treatment plans, fractionation schemes, total dose, biologically equivalent dose, patient characteristics, tumor location, medications and other agents, surgical history, radiation sensitivities of the tumor and normal tissue, and patients’ underlying medical status. Some late effects seen with PT could be attributed to the use of previous technologies (eg, passive scattering proton therapy [PSPT]). PT has now evolved to intensity modulated proton therapy (IMPT) and provides superior dose conformity and lower doses to the normal tissue near target volumes, but its complexity and temporal spot delivery path may pose challenges in the delivery of dose and its distribution within the target.^17^ Figure 1 shows the details of trials conducted with PT. As of December 2020, ClinicalTrials.gov has registered 180 trials in “proton therapy”: 78 studies recruiting, 31 completed, 26 active but not recruiting, 15 terminated, 11 not yet recruiting, and 11 withdrawn (Fig. 1A). The percentage of trials for different cancer types is shown in Figure 1B.

A summary of the results of the 3 completed randomized trials with results available comparing PT with photon therapy is provided in Table 1.

### SBPT versus SBRT for high-risk, early stage NSCLC (NCT01511081)

This randomized phase 2 study compared stereotactic body RT (SBRT) with stereotactic body proton therapy (SBPT) for high-risk, medically inoperable, early stage, centrally located, stage I, II, and recurrent non-small cell lung carcinoma (NSCLC) for side effects, quality of life (QoL), and cancer control.^18^ The experimental arms SBRT and SBPT both received 50-Gy (relative biological effectiveness [RBE]) in 4 daily treatments. One patient in the SBPT group developed grade 3 skin fibrosis, and no patient experienced grade 4/5 toxicities. No serious or other (excluding serious) AE was reported in both arms. Due to low accrual, no meaningful comparison could be made for efficacy and toxicities.^18^

### PSPT versus IMRT for locally advanced NSCLC (NCT00915005)

This phase 2, randomized, open-label trial compared the toxicity and effectiveness of PSPT with that of standard IMRT, both with concurrent chemotherapy (carboplatin or paclitaxel), for patients with locally advanced NSCLC (stage II-IIIB, stage IV disease with single brain metastasis, or recurrent tumor after surgical resection).^19^ The primary endpoint was the first occurrence of severe (grade ≥3) radiation pneumonitis (RP) or local failure (LF). With the Bayesian adaptive design, of the 275 enrolled patients between 2009 and 2014 at the UT–MD Anderson Cancer Center and Massachusetts General Hospital, 92 and 57 patients were treated with IMRT and PSPT, respectively. Patients underwent standard treatment planning, with 4-dimensional computed tomography (CT) for motion assessment and target delineation and dosimetric comparison for IMRT and PSPT.

In both plans, the prescribed tumor dose was 74 or 66 Gy (RBE), whichever could be safely achieved according to the specified dose constraints. The tested hypothesis was that PSPT exposes less lung tissue to radiation than IMRT and thereby reduces toxicity. Table 2 provides the mean dose in Gy to critical organs at risk (OARs), showing no significant difference between IMRT and PSPT to the lung and esophagus and a significant reduction in heart dose with PSPT. RP was scored with the Common Terminology Criteria for Adverse Events, version 3.0 (CTCAE 3.0). At the median follow-up time (24.1 months for IMRT and 25.7 months for PSPT for all patients), 12 patients developed grade ≥3 RP (6 in each group). At 1 year, 2 patients in the IMRT group had grade 4 or 5 RP, and no patients in the PSPT group had grade 4 or 5 RP. There were no differences in the rates of LF. Thus, although PSPT did not improve dose-volume indices for the lung, there was no benefit noted in RP or LF after PSPT; however, improvements in both endpoints were observed over the course of the trial.^19^

**Fig. 2.**
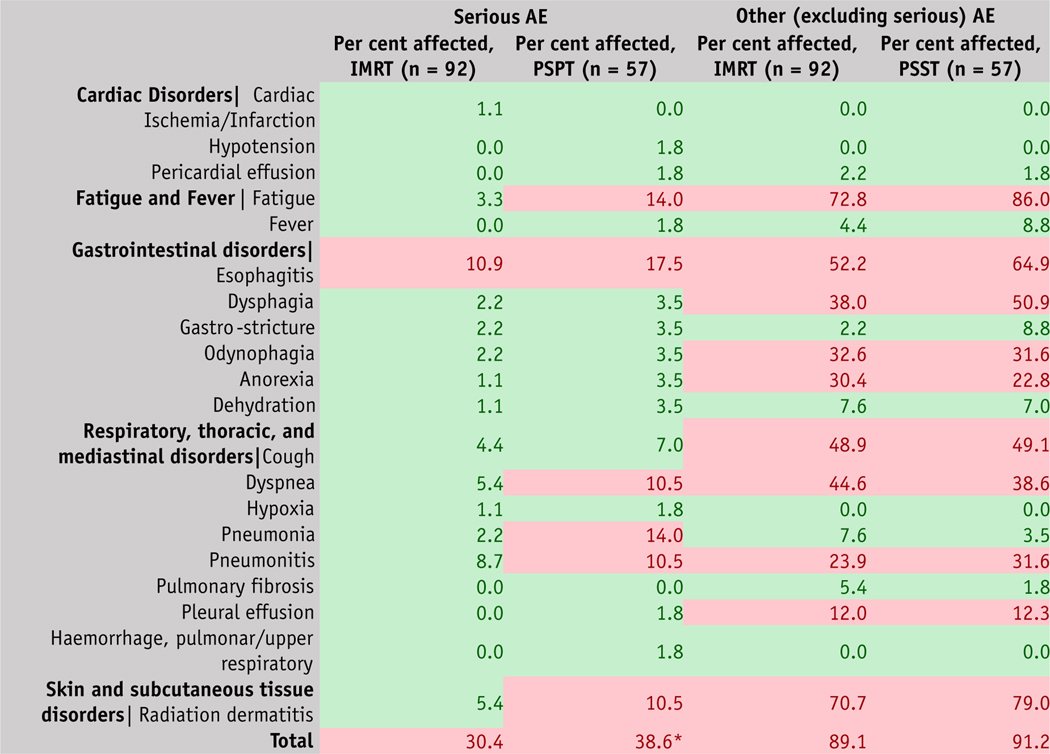
Adverse events (AEs) in clinical trial NCT00915005, as reported in clinicaltrials.gov, with vocabulary Common Terminology Criteria for Adverse Events, version 4.0. The percent total of all events is not the percent of numerical total, but rather the number of events collected by systematic assessment. Several AEs can occur together in a given patient. To test the statistical significance between variables, a 1-tail *t* test was used assuming unequal variances between 2 samples. All serious AEs (grade ≥3) were significantly higher in the passive scattering proton therapy group compared with intensity modulated radiation therapy (*P* <.01), but other AEs (excluding serious) were not significantly (*P* <.40) different between the 2 groups. Conditional formatting in Microsoft Excel in a 2-color scale is used to depict percent AEs, with cells that contain values >10% shown in red and <10% in green. * Serious AEs are significantly higher (*P* < .01) in PSPT group compared to IMRT

For comparison of serious (grade ≥3) and other AEs, we used subsequently updated data from this trial (March 26, 2020) at clinicaltrials.gov (https://clinicaltrials.gov/ct2/show/results/NCT00915005). AEs recorded in accordance with the CTCAE 4.0 in ClinicaTtrials.gov indicated that all serious AEs (grade ≥3) were significantly higher in the PSPT group (*P* < .01), which was due to a numerical increase in several AEs, including fatigue and fever; gastrointestinal (GI) disorders (eg, esophagitis, dysphagia, and odynophagia); respiratory, thoracic, and mediastinal disorder (eg, cough, dyspnea, hypoxia, pneumonia, pneumonitis, pleural effusion, hemorrhage, pulmonary/upper respiratory); and skin and subcutaneous disorders (radiation dermatitis), compared with IMRT (Fig. 2). However, other AEs (excluding serious) were not significantly (*P* < .40) different between the 2 groups.

The higher incidence of serious AEs in the PSPT group may be due to several reasons. First, a higher RBE of the protons, especially in the lateral penumbra and at the distal end of the Bragg peak, resulted in a higher-than-expected-dose to OARs, the esophagus, and the lung than the RBE used in treatment planning (1.1).^20^ The range of estimated doses to these OARs in both the treatment groups was similar. Minimizing the dose to the heart might have been essential to reduce the risk of cardiac disorders in the PSPT group. Second, organ motion due to breathing resulted in exposure of the normal lung volume and affected the outcome; there are risks with all conformal modalities, and motion mitigation is perhaps even more important for protons.^21^ In addition, Liao et al.^19^ rightly noted and suggested the presence of a learning curve in the delivery of PSPT, which was observed with corresponding improvements over the course of the trial in the PSPT group. Finally, robustness has only recently become a mandated component of clinical trials. Robustness is present in photon plans but needs to be added to proton treatment plans to address the additional uncertainties noted here with mathematical, reproducible formality.^22,23^

Of note, a recent comparison of toxicity profiles and survival after IMPT versus PSPT for NSCLC suggested that IMPT lowers radiation doses to the lung, heart, and esophagus with concomitant lower cardiopulmonary toxicities (grade ≥3).^24^ These results confirm our conjecture that with technological improvements in dose delivery and distribution, normal tissue toxicities could be significantly reduced. Further improvements in the reduction of AEs in the treatment of lung cancers may also be achieved with unform active scanning to overcome some of the challenges discussed herein.^25^

### Proton beam therapy versus IMRT for locally advanced esophageal cancer (NCT01512589)

This phase 2, randomized, open-label, single-institutional trial compared the total toxicity burden (TTB) and progression-free survival (PFS) of proton beam therapy (PBT) to IMRT (both with concurrent chemotherapy) for patients with locally advanced esophageal cancer.^26^ After randomization, all patients received concurrent chemotherapy (fluorouracil and capecitabine with a taxane, or carboplatin with a taxane, or fluorouracil with oxaliplatin; patients eligible for esophagectomy received resection 8–10 weeks after conformal RT) and RT (50.4 Gy in 28 fractions) with individualized treatment planning. The primary outcome measures were PFS (timeframe 6 weeks after RT) and TTB (timeframe 12 months after RT), which was computed as a composite score from serious AEs (CTCAE 4.0) and among patients who underwent surgery for postoperative complications (POC). Secondary objectives included a description of physician-assigned toxicities, defined by CTCAE 4.0 and patient-reported QoL. With the Bayesian group sequential design, of the 145 enrolled patients between 2012 and 2019, the evaluable patients included 61 treated with IMRT and 46 with PBT. Approximately 48% of patients (28% IMRT and 20% PBT) underwent esophagectomy.

The mean TTB was 2.3 times higher for IMRT (39.9; 95% highest posterior density interval, 26.2–54.9) compared with PBT (17.4; 10.5–25.0), and for the surgical population, the mean POC score was 7.6 times higher for IMRT (19.1; 7.3–32.3) versus PBT (2.5; 0.3–5.2). The PBT arm experienced numerically fewer POCs and toxicities, which included cardiopulmonary toxicities, atrial fibrillation, asymptomatic effusions, lower-grade radiation pneumonitis, and acute respiratory distress syndrome. Furthermore, PBT also reduced grade 4 lymphopenia compared with IMRT. For patients who completed conformal RT, there were 3 grade 5 events, all in the IMRT group. One patient died 47 days after surgery as a result of acute respiratory distress syndrome, pneumonia, and sustained atrial fibrillation. Two patients who did not undergo surgery died 12 and 15 days after completing conformal RT as a result of pulmonary embolism and myocardial infarction, respectively. The 3-year PFS rate (50.8% IMRT vs. 51.2% PT) was not different between the 2 groups, and the 3-year overall survival (OS) rates (44.5% vs. 44.5 %) were similar. Thus, for locally advanced esophageal cancer, PBT reduced the risk and severity of AEs compared with IMRT while maintaining similar PFS and OS.

This trial clearly demonstrates that the dosimetric advantages of PT over IMRT can translate to reduced TTB and POC in locally advanced esophageal cancers. As discussed by Lin et al,^26^ the fewer POCs seen may be due to the lower integral dose, and the effect of organ motion is less pronounced for esophagus compared with the lungs. Furthermore, PBT delivers a dose to a substantially lower volume, and the lymphocyte sparing effect could also be more pronounced.^27^

### Organs at Risk, Adverse Events, and Other Toxicities

This section is introduced by critical issues related to the use of PT, followed by reported observations from the treatment of cancers of various organ sites. Based on the published conclusions, the authors generally present the mostly qualitative data on the comparison between photon and PT therapies.

#### Gastrointestinal cancers

PT appears to reduce the exit dose to the heart and lungs during the treatment of esophageal cancers. Neoadjuvant PT may reduce the incidence and severity of pulmonary, cardiac, and GI toxicities.^28^ The common grade 2 to 3 acute toxicities observed with concurrent chemotherapy with PT in a prospective study were esophagitis, fatigue, nausea, anorexia, and dermatitis, aside from pneumonitis, although this study was based on dosimetric advantages rather than a direct comparison with photon therapy.^29^ Similarly, the study that compared treatment planning between PT and IMRT in gastric cancer suggested significantly lower rates of acute and late toxicities to the small bowel, liver, kidneys, and heart with PT.^30^ In the treatment of hepatic cancers, the risk of radiation-induced liver disease correlates with normal tissue dose and limits the curative doses due to the low tolerance of the liver.^31,32^

For pancreatic cancers, PT may improve the therapeutic index because proton plans indicate lower doses to the kidneys, stomach, liver, and bowel.^33^ Preliminary data from the Massachusetts General Hospital (Boston, MA) and the University of Florida (Jacksonville, FL) in the United States, as well as the Hyogo Ion Beam Centre (Hyogo, Japan), have become available. A phase 1/2 study of postoperative short-course chemoradiotherapy with PT and capecitabine, followed by early surgery for resectable pancreatic ductal adenocarcinoma, showed that the treatment was well tolerated and resulted in excellent local control.^34^ A phase 1/2 study of chemoradiotherapy with gemcitabine with concurrent PT for most patients (67 Gy, RBE in 25 fractions) with locally advanced pancreatic cancer in Japan showed a high rate (45 of 91 patients) of stomach and duodenum ulcers,^35^ although initial reports indicated tolerability of this regimen.^36^ PT with concomitant capecitabine for the treatment of marginally resectable and unresectable/inoperable pancreatic and ampullary adenocarcinoma (PT doses 50.4–59.4 Gy, RBE) at the University of Florida showed no grade 3 toxicities.^37^ Further improvements in systemic therapy are crucial for PT to make a bigger impact on cure in the postoperative setting,^38^ because exacerbation of toxicities is a common problem with radiation–drug combinations.^39,40^

#### Breast cancer

Recently reported data on the single-group, open-label, prospective trial evaluating the effectiveness of 3-dimensional conformal PT or IMPT in invasive breast cancer indicated significant serious AEs, including dermatitis and skin fibrosis, as well as other AEs, including anemia, dermatitis, pneumonia, and pulmonary fibrosis (NCT01340495), although early results (4 and 8 weeks after completion of therapy) among 12 patients with breast cancer were favorable.^41^ A comparison of heart doses for breast cancer with PT and IMRT suggested that PT lowers the average mean heart dose (2.6 Gy) compared with IMRT (5.6 Gy).^42^ PT may be advantageous to reduce the cardiovascular dose when internal mammary nodes are included in the target volume^43^ and mitigating the higher risk of heart exposure associated with the tumor location on the left breast may be feasible with PT with the deep breath-hold technique to keep such doses to <1 Gy.^44^

A comparison of dosimetric studies that applied the normal tissue complication probability (NTCP) model among patients with tumors in the left breast indicated that with IMPT, significant heart and lung dose sparing could be achieved compared with IMRT.^45^ The risk of subsequent ischemic heart disease, which is proportional to the mean heart dose, can be evident after a few years and continue for decades.^46^ Preexisting cardiac risk factors can further increase this risk, although the absolute excess risk of cardiac morbidity seems to be less with protons (0.13%) compared with photons (1.0%).^47^ Therefore, a combined assessment of the risks from preexisting conditions, cardiac exposures, and inadequate target coverage, as well as long-term follow-up, is essential for a systematic comparison of photon versus PT plans. RadComp (https://www.radcomp.org/) is now evaluating the differences between PT and photon therapy for cardiac dose distribution related to reductions in cardiac morbidity, mortality, QoL, and cancer control outcomes for nonmetastatic breast cancer in a large-scale randomized trial.^48^

#### Hodgkin lymphoma

A comparison of normal tissue doses calculated for 21 patients with Hodgkin lymphoma treated with pencil-beam scanning PT versus 3-dimensional conformal RT and partial volumetric modulated photon RT indicated that the former significantly reduced the mean dose to the heart, breast, lungs, spinal cord, and esophagus; increased dose homogeneity and conformity within the target volume; and provided dosimetric benefits for patients whose clinical treatment volume (CTV) extended below the seventh thoracic level and female patients with axillary disease.^49^ A comparison of helical tomotherapy and PT with standard 3-dimensional conformal RT in 14 female patients with supradiaphragmatic Hodgkin lymphoma showed significantly lower mean doses to the breasts, lung tissue, and heart. Interestingly, helical tomotherapy achieved better protection of the lungs at doses >15 Gy than passive PT or 3-dimensional conformal RT; however, dose distributions could generally be improved even further by pencil-beam scanning PT.^50^ These studies indicate that patient selection based on the anatomic location of the tumor can reduce normal tissue injury in PT.

#### Prostate cancer

Long-term posttreatment QoL is an important issue in prostate cancer treatment. A systematic review of the literature and a meta-analysis of 5 randomized clinical trials (RCTs) showed that treatment with IMRT or PT can significantly decrease the severity of both moderate and severe late GI toxicities and may allow dose escalation.^51^ Five-year clinical outcomes with image guided PT indicated high efficacy, minimal physician-assessed toxicity, and good patient-reported QoL outcomes, although a larger patient experience is essential to confirm these outcomes.^52^ A comparison of patient-reported QoL collected at a single center during the first 2 years after treatment in a large cohort of 1243 men (PT doses 76–82 Gy) and 204 men (IMRT doses 75.6–79.4 Gy) showed no significant differences in bowel toxicities, urinary incontinence, and urinary irritative/obstructive and sexual domains between the 2 cohorts.^53^ However, men who received IMRT reported moderate/big problems with rectal urgency and frequent bowel movements compared with those who received PT during the early follow-up of up to 2 years. These outcomes suggest the need for longer follow-up because these toxicities occur late.^53^ Similarly, a prospective comparison using a case-matched analysis for risk groups, age, and prior GI and genitourinary disorders showed no statistically significant differences in the risk of grade ≥2 acute and late GI and genitourinary toxicities between 213 patients treated with IMRT to 79.2 Gy and 181 patients treated with PT to 79.2 Gy (RBE).^54^ Although some prospective studies appear to show the safety and efficacy of PT, no studies have unequivocally demonstrated a definite benefit of protons over IMRT; therefore, long-term prospective studies with larger patient cohorts are necessary to analyze the effectiveness and reduction in long-term toxicities.^55^

#### Lung cancers

Both NRG and the Particle Therapy Cooperative Group, Thoracic Subcommittee task group are addressing the issues of PT indications, advantages and limitations, cost effectiveness, technology improvement, clinical trials, and future research directions in the treatment of lung cancers.^56^ In the treatment of lung cancers, organ motion is a critical issue that affects treatment quality. In addition to the geometric blurring of dose gradients, target motion during treatment planning causes uncertainties in the dose to both the tumor and normal tissue, as well as its distribution. The sources of interferences that cause uncertainties include interfield motion in IMPT and interplay between scanning and intrafractional (or intrafield) motion, which require stringent guidelines for motion monitoring and motion mitigation.^57^ Throughout the thoracic region, intrafractional changes are unavoidable due to respiration and cardiac motion. Hypofractionation treatment of moving targets with scanned beams for motions of amplitudes >5 mm require motion mitigation strategies.^58,59^

The pros and cons and clinical status of various motion management techniques, such as treatment during breath hold, rescanning, gating, or tracking, has been recently reviewed, and robustly optimized treatment plans might be a clinical solution in the treatment of lung cancers.^57^ For beam scanning in IMPT, the worst-case dose distribution can mitigate the influence of uncertainties, which is obtained by assigning the lowest dose among several doses to each voxel in the CTV and the highest dose to each voxel outside the CTV to provide robust target coverage without sacrificing or improving the sparing of normal tissues.^60^ Exploratory studies comparing the impact of uncertainties and interplay effect on 3- and 4-dimensional robustly optimized IMPT treatment plans for lung cancer showed that 4-dimensional robust optimization methods produced significantly more robust and interplay-resistant treatment plans for targets with comparable dose distributions for normal tissues.^61^ In IMPT plans, incorporating the changes in anatomy caused by respiratory motion, along with setup and range uncertainties, into the 4-dimensional robust optimization system was tested in a small number of patients with lung cancer, and showed the potential to improve target and normal-tissue dose distributions.^22^ A recent study with proton-based 4-dimensional robust SBRT for patients with early stage NSCLC demonstrated that degradation of the target dose distribution associated with interplay effects could be mitigated by using iso-energy layer repainting techniques.^62^ However, a common framework applicable across different treatment techniques and modalities to conduct a robustness analysis is essential, and an overview of important elements required for the unambiguous reporting of uncertainty scenarios and their dosimetric effects was recently described.^23^

Randomized data comparing clinical outcomes between proton and photon radiation are limited to a small number of studies.^18,63^ Two examples were discussed previously (NCT00915005 and NCT01511081). As newer PT techniques (eg, IMPT) are increasingly used along with increasingly available immunotherapies, tumor control should improve, and these factors should result in a significant reduction in toxicities in the treatment of lung cancers.^63^

#### Tumors of central nervous system and brain

Multiple dosimetric studies support the use of PT in benign and low-grade pediatric central nervous system (CNS) tumors.^64^ However, for certain types of tumors, such as vestibular schwannoma, proton data seems to be inferior to advanced therapies with photons at the present time.^65^ Among 313 children treated with PT to doses >50.4 Gy (Cobalt Gy equivalent) for ependymoma, craniopharyngioma, and lower-grade glioma, the 2-year cumulative incidence of brain stem toxicity was 3.8% ± 1.1%, and grade 3+ toxicity was 2.1% ± 0.9%.^66^ Tumor location (supratentorial) and the extent of surgical resection (non-gross total resection) were negative prognostic factors for both OS and PFS with no decrease in QoL among children treated with pencil-beam scanning PT for atypical teratoid/rhabdoid tumors.^67^ The most common late toxicity was endocrinopathy (45%) after PT for CNS germinomas or nongerminomatous germ cell tumors among 20 children who received PT between 2006 and 2009, with a median follow-up time of 5.6 years.^68^ Radiation necrosis in eloquent areas of the CNS can also occur as early as 3 months and as late as 13 years after RT.^69^

PT could be used to spare critical structures that are important for cognitive development, endocrine function, and hearing preservation and to reduce the total body dose and second malignancy risk.^64^ The risk of cognitive impairment decreases with the mean hippocampal dose and decreasing treatment margins.^70^ Recently published data on the change in intellectual scores over time in pediatric patients with medulloblastoma suggested favorable intellectual outcomes in most domains with protons compared with photons, although processing speed was a vulnerable domain for both treatment groups.^71^ A myriad of late effects after PT may also be due to additional vascular toxicities detected with imaging.^72,73^ The average rate of symptomatic brain stem toxicity was 2.38% among 671children with focal posterior fossa tumors treated with protons. The actuarial rate of grade ≥2 brain stem toxicity was successfully reduced from 12.7% to 0% at 1 center after adopting modified radiation guidelines for treatment planning after consensus brain stem constraints for PT.

The established guidelines take into consideration substantial dosimetric data collected for brain stem injury after PT to allow safe delivery of radiation. The National Cancer Institute conducted a workshop in May 2016 to examine brain stem toxicity among pediatric patients treated with PT to indicate that opportunities still exist to incorporate the optimization of linear energy transfer (LET). Doing so requires research to exploit the capabilities of LET- and RBE-based planning.^4^ A more conservative brain stem dose limit with protons is now in use in cooperative group trials to reduce necrosis and other toxicities, but it is still short of analyzing and addressing biologic issues, such as LET and RBE optimization. More sophisticated plan evaluation approaches will likely be needed to properly address and replace this simplistic approach.^74,75^ For skull base meningiomas, data from stereotactic series and IMRT show excellent local control with minimal side effects. Thus, any improvement with protons might only be marginal. RCTs with long-term follow-up for toxicities are still needed to establish the superiority of protons in the treatment of CNS tumors.^65^ Some toxicities could be due to failure to fully optimize the PT, not just for the Cobalt Gy equivalent dose, but also for the variation in the dose due to LET and ultimately for RBE due to variation in tissue sensitivities to the delivered dose.

#### Head and neck cancers

Several retrospective cohort comparison studies have indicated the benefits and suitability of PT in the treatment of head and neck cancers (HNC) over photons in terms of dosimetric advantages, translating into improved acute toxicities.^76–80^ Prospective randomized studies are necessary and ongoing (eg, NCT02923570 and NCT01893307). A comparison of IMRT and IMPT treatment plans for acute mucositis, xerostomia, aspiration, dysphagia, laryngeal edema, and trismus to determine whether a subpopulation of patients with head and neck squamous cell carcinoma could benefit from PT indicated a general reduction in NTCP values while keeping similar target coverage.^81^ The risk reduction for acute mucositis was more pronounced in patients with tumors in the larynx region, which suggests that tumor location-based patient preselection may provide better outcomes with IMPT.^81^

A quantitative clinical decision-support strategy to identify the patient population that would benefit most from PT suggested that younger patients with p16 positive tumors and who smoked less were estimated to have better quality-adjusted life years with PT compared with IMRT.^82^ PT plans had lower doses to the brain stem, spinal cord, oral cavity, contralateral parotid, and contralateral submandibular regions compared with IMRT plans in the treatment of major salivary gland cancer or cutaneous squamous cell carcinoma, resulting in significantly lower rates of grade ≥2 acute dysgeusia, mucositis, and nausea.^77^ Similarly, patient-reported outcomes with IMPT for oropharyngeal cancer showed a lower symptom burden compared with IMRT.^78^ CTV-based robust optimization of PT plans can minimize exposure of OARs and achieve robust dose distributions to targets compared with IMPT without such optimization.^83^ Such optimization could form the basis for subgrouping of patients based on human papillomavirus infection status for dose modification with IMPT or IMRT to limit toxicities.

#### Blood as organ at risk

Circulating lymphocytes are a radiosensitive cell population, although subpopulations show differences in radiosensitivity.^84^ Combining chemotherapy with radiation is often required in the treatment of a variety of cancers but can exacerbate lymphopenia and cause immunosuppression. A low baseline lymphocyte count across a range of cancer types has traditionally been a negative outcome predictor.^85^ Age, planning treatment volume with body mass index, baseline absolute lymphocyte count, and radiation treatment modality (PT or IMRT) are factors influencing lymphopenia and thereby PFS and OS.^86^ Grade 3 and 4 lymphopenia occurs in the majority of patients treated with chemoradiotherapy.^87^ Therefore, of late, blood is seen as a moving OAR, and lymphocyte-sparing RT has been advocated to mitigate treatment-related lymphopenia.^88^ Lymphocyte-sparing RT aims to deliver a dose as low as reasonably achievable to lymphocyte-rich regions, including large blood vessels, the heart, and lymphoid organs.^88^ Irradiating with PT, hypofractionation, or radio-surgery in the setting of patients with NSCLC reduced lymphopenia, which is a prognostic indicator in patients receiving immunotherapy.^89^ Among patients with esophageal cancer treated with definitive chemoradiation, a retrospective analysis indicated that PT reduced the risk of sustained grade 4 lymphopenia compared with IMRT,^90^ and a reduction in integral dose reduced grade 4 lymphopenia with PT.^27^ A 4-dimensional human blood flow model to estimate the dose to the circulating blood during intracranial treatment was also developed, which could potentially be used to stratify patients to proton- versus photon-based therapies.^91^ A direct causal relationship between lymphopenia and poor survival is likely but remains uncertain until interventional studies are conducted to show improved outcomes with lymphocyte-sparing RT. With the recent interest in combining immunotherapy with RT, future preclinical and clinical studies should elucidate the impact of lymphopenia on immunotherapies. Future efforts should also encourage optimal patient enrollments and retention, with an emphasis on RCT, and the results should be evaluated for the comparative effectiveness of PT.^92^

Most studies that compare PT with other RT modalities demonstrate the benefit of NTCP reduction with PT over photon therapies in treatment planning. However, its clinical benefit is yet to be established in prospective RCTs in the treatment of cancers of several organs/sites. To fully benefit from PT, the design of future trials should be geared toward the personalization of treatments based not only on dosimetric advantages of PT but also on biological determinants such as RBE, tumor response, individual sensitivity, and a foundational understanding of radiobiological differences between these 2 radiation types in determinants of normal tissue injury.

### Biological Dose–The Missing Factor

A steady increase in PT literature between 2010 and 2020, with a yearly breakdown and Figure (Supplement) summary statistics of the published literature are provided in Figure (Supplement). Of the 4807 published articles, 3974 were original research articles and 833 were others (reviews, meeting reports, and conference proceedings). Figure (Supplement)

#### Biophysical Issues

Known issues and contributing factors that cause normal tissue injury with PT are provided in Box (Supplement).

#### Uncertainties in the depth of penetration

Along the entrance path to the tumor, proton beams deliver relatively low doses and deposit most of their energy over a narrow field at the distal end, called the Bragg peak. Tissues along the exit path are also expected to receive a negligible dose. The uncertainties in the depth dose profile and dose conformity due to a shift in the Bragg peak position, owing to differences in tissue composition, movement of the target, and breathing motion, have been reduced with advances in 4-dimensional CT-based motion management and IMPT.^93^ Because PT is more precise and less forgiving for normal tissue injury, it is important to delineate the target volume even more accurately, for which a high degree of image guidance is crucial.^94^ In this context, volumetric imaging by cone beam CT is an alternative to routine CT-based image guidance and has become available for monitoring anatomic changes, assessing delivered dose, and adapting planning during treatment in the setting of adaptive PT.^95,96^ A cone beam CT-based adaptive PT workflow in a retrospective clinical investigation of 20 patients with lung cancer provided clinical indicators comparable to those using rescan CT.^95^

#### Relative biological effectiveness: Linear energy transfer, dose, fraction, and tissue type

RBE values of protons of 1.1 to 1.2 used at the clinic are mostly derived from cell culture data; however, animal-based data are likely superior.^97^ Factors such as LET, dose/fraction, and tissue type determine RBE. The effect of protons proximal to spread-out Bragg peaks are similar to the effects of photons; however, at the distal end, the quality and quantity of damage by protons (eg, DNA damage) will be different. The type of DNA damage has implications for injury manifestation both in tumors and normal cells.^98^ A higher RBE, if incorporated into treatment planning, may allow for better tumor control with protons, but it can also overdose normal tissue; therefore, these issues should be taken into consideration during treatment planning to minimize normal tissue injury. Disregarding RBE variations for various fractionation schedules using the published RBE models and α/β assumptions indicated that model predicted RBE values differ substantially from 1.1 in the treatment of prostate cancer, and this variation is more pronounced at a standard fractionation relative to hypofractionation.^99^ For personalization of PT to reduce normal tissue injury, the use of variable RBE is necessary.^6,100^ Novel mathematical models that can estimate the RBE from the dose, LET, and different fractionation regimens have become available.^101^

#### Delineation of target volume

Image guidance with CT/magnetic resonance imaging–based planning enables accurate delineation and contouring of the tumor, but CT-based imaging has limited resolution in defining tumor margins and extended tumor infiltration beyond the visible margin of the primary tumor or lymph nodes. Because of the uncertainties in CTV definition, PT with a sharp dose falloff beyond the gross tumor volume may lead to significant underdosing of the tumor and unintended higher dose to the normal tissue.^94^ The use of sharp dose gradients may compromise target coverage or even geographically miss the tumor altogether.^102^

#### Target and organ motion

A higher degree of accuracy in dose delivery to the target is essential to maintain dose conformality for PT. Imaging capabilities in many PT facilities are less sophisticated compared with photon facilities.^103^ Four-dimensional optimization techniques have been envisioned to be excellent solutions for motion mitigation.^92^ Positron emission tomography/CT scans can image metabolically active tumors, but the inability to track organ motion is a confounder for positron emission tomography/CT–based imaging. A tracking method that exploits artificial neural networks to estimate the internal tumor trajectory as a function of external surrogate signals could be a valuable tool for accurate real-time tumor tracking.^100^ The technical difficulties in PT to overcome motion management and inhomogeneity of tissues, especially along the exit path of protons, might expose a large volume of normal tissue, resulting in the loss of any gain obtained with the improved dose distribution.

### Biological issues

#### Differences in cellular injury between protons and photons

An understanding of mechanisms of cell killing is necessary for and fundamental to designing future trials geared toward accurate precision RT with protons; however, the mechanisms are far less understood in comparison with photons.

Monte Carlo track structure simulations indicate significant differences in the microscopic distribution of energy among different radiation types.^104^ The frequencies of energy loss by electron interactions increase approximately from 3% for 10 keV to 77% for 300 MeV protons.^105^ Protons within or near the Bragg peak will cause clustered DNA damage, which is quantitatively and qualitatively different and refractory to DNA repair.^106^ Other cellular moieties are also important critical targets. Microdosimetry could be used to characterize the radiation quality factor at the Bragg peak, which can vary with the size of the target volume and have a strong bearing on the RBE peak.^98^ Furthermore, there are differences due to the high LET effects of protons at the end of the range and in the beam penumbra in addition to, for example, inelastic nuclear collisions that protons undergo, which might also induce significant biological consequences and are quite different from high-energy photons.^105^

The amount of clustered DNA damage induced varies with radiation type.^107^ The frequencies of complex double-strand breaks (DSBs) increase with LET and dose. Furthermore, clustered DNA damage can lead to the induction of additional DSBs.^98^ When clusters of complex DNA lesions involve ≥2 lesions in proximity in time and space, the repair is less efficient. The effects of radiation quality and hypoxia on the induction of clustered damage have been previously reviewed; both yield and spatial distribution of DSBs appear to be nonrandom.^108^ Protons are more efficient in producing cytogenetic damage than x-rays in human peripheral blood lymphocytes, which may be also due to a difference in the intracellular distribution of energy and differences in energy itself.^109^ Furthermore, protons and photons induce cell killing by different modes,^110^ which has implications for the potential of the given radiation type to cause immune modulation via programmed cell death (x-rays) or necrotic cell death (PT).^110,111^

DNA damage and DNA damage response (DDR) pathways have been previously reviewed.^112^ Many details on the induction and processing of clustered DNA damage, near or within the Bragg peak induced by PT, have not been elucidated. Nonhomologous end joining seems to be critical in the DDR to photon irradiation,^108^ but homologous recombination appears to be more important for the repair of particle-induced clustered DNA damage.^6^ The repair of complex DSBs will be slower due to the necessity of chromatin decondensation for the repair to occur, compared with the repair of simple DSBs. Although the average repair time for simple and complex DSBs does not vary with radiation quality, the relative proportions of these DNA lesions will depend on radiation quality.^98^ Differences with LET in the induction of DNA damage and its distribution can be studied at the microscopic level by premature chromosome condensation,^113^ gamma-H2AX foci,^114^ and micronuclei^109^ assays. Spatial distribution of DSBs induced by charged particles in a mouse model suggest an inhomogeneous distribution compared with photons.^115^ The distribution of micronuclei in human peripheral blood lymphocytes indicates an overdispersion after irradiation protons compared with photons.^109^ The biological response induced by protons differs for several other endpoints compared with photons (eg, angiogenesis and cell migration), as well as the induction of secondary cancers.^116^ Proton irradiation (0.5–2 Gy) downregulated some genes in a dose-dependent manner, in contrast to a dose-dependent upregulation observed after photon irradiation.^116^ Even with photons, when compared with a single dose, the exposure to multifractionated radiation results in transcript and miRNA expression differences in terms of number and magnitude.^117^ Furthermore, differential gene expression with protons might elicit different responses in cell-cycle regulation and DNA repair.^118^ Many tumors have defects in DDR pathways, which can be exploited to increase the therapeutic ratio of PT and personalize RT. Thus, differences in microdosimetric spatial distribution of energy, DNA damage and repair, and gene expression changes between radiation types may have implications on the potential of a given therapeutic modality, used alone or in combination with other agents, to cause a difference in biological response (eg, immune suppression vs. modulation).^110,111^

#### Patient-related factors

Tumor provides a highly spatially and temporally dynamic environment to the normal tissue because of ever-changing tumor characteristics and therefore can predispose normal tissue to AEs. The quality of the radiation used can affect the tumor’s biology and interactions with the host. For example, there is a significant difference in cellular proliferation after irradiation with protons and photons.^109^ Differences in the response of a tumor to a given radiation type in light of the role of the tumor itself on normal tissue injury is an area that has not been adequately pursued, but these factors are beyond the scope of this paper.

### Biomarkers

Noninvasive monitoring of tumor response and normal tissue toxicities is necessary for personalization of RT,^16,119^ including PT. Biomarkers or panels of biomarkers that could predict AEs before, during, or after RT would be useful. Of note, an integrated digital suppression method to overcome the historical barriers on the use of circulating tumor DNA (eg, insufficient quantities of cell-free DNA and sequencing artifacts) that limit the analytic sensitivity by eliminating background artifacts and molecular bar-coding for the efficient recovery of cell-free DNA molecules has been described.^120^ Similarly, a method to dynamically determine outcome probabilities for individual patients using multiple risk predictors acquired over time (ie, continuous individualized risk index) has also been described.^121^ An analysis of cell-free RNA molecules in plasma may allow noninvasive assessment of gene expression changes related to inflammation in a variety of tissues and cell types.^122^

### Conclusions and Future Directions

In general, dose prescriptions to tumors are based on the tumor control probability and NTCP; both are based on a population average for a given organ/site. However, the response of the tumor, as well as surrounding normal tissues and individuals, to radiation varies widely across a population. RT is a highly personalized treatment approach because it takes into consideration anatomic features and patient characteristics in treatment planning.

An analysis of recently completed randomized trials indicated that PSPT (which is now largely replaced by spot-scanned IMPT) did not provide any additional benefit in reducing normal tissue toxicities or locoregional failure for locally advanced NSCLC (NCT00915005).^19^ However, PBT provided a lower total toxicity burden in patients with locally advanced esophageal cancer, although it did not improve PFS and QoL (NCT01512589).^26^ The results from the few comparative studies of efficacy and AEs have limitations considering that advances in technology, physics, and biology are a continuum; hence, the process of optimal proton therapy delivery needs to be seen as an iterative process in a defined path. We conducted a head-to-head comparison of different areas of proton versus photon therapy; thus, some degree of uneven presentation is unavoidable due to advancements of technologies and data. Nonetheless, for the appropriate clinical use of PT and justification of costs, comparative trials are essential, recognizing that ongoing development is a reality with almost every medical intervention, and well-done data at the extant state-of-the-art/science are essential for further advancement. A summary of issues and future directions in the comparisons of proton therapy with photon therapy in the treatment of cancers of various organ/sites is provided in Table 3.

Most studies with PT used retrospective analysis and single-arm studies will likely favor PT; therefore, prospective, large-scale, well-designed RCTs are necessary.^48^ To fully benefit from PT, in our opinion, the design of future trials should be geared, when possible, toward accurate precision RT incorporating suitable biomarkers,^16^ which is necessary for and fundamental to optimizing the advantages gained due to the mechanistic differences between protons and photons in inducing damage, both in the tumor and normal tissues.

Collateral normal tissue injury seems inevitable with either type of radiation treatment. Yet, there is a clear difference between photons and protons in how they interact with tissue, with significant biological implications. There is an unmet need to better understand dose-volume effects of radiation beyond the established normal tissue NTCP models to guide optimization of dose distributions, reduce the incidence of normal-tissue toxicities, and allow selection of the optimal tumoricidal dose of radiation at the individual patient level.^123^ Protons and heavier charged particles beams are distinct from photons, not only concerning their unique dosimetry, but also in their ability to invoke unique biological responses that are differentially exploitable. Therefore, continued studies on the biology of these differences are necessary in designing next-generation trials to compare and benefit from a given type of radiation treatment. RT dose is not only physical, but also biological; however, physical dose is only one part of the total dose a patient sees when undergoing treatment for cancer.^16,123^ Thus, clinical trials in PT need to be optimized, addressing the differential biological complexities, and such trials could include biological dose concepts to properly answer the question of which treatment is better or worse for a given patient or patient cohort.

### Search Criteria

For this review, we obtained data from 2 publicly available databases: U.S. National Library of Medicine’s ClinicalTrials.gov (https://clinicaltrials.gov) for the analysis of clinical trials, and the PubMed database using iCite (https://iCite.od.nih.gov/), a web application to access data on published clinical trials and bibliometric information for publications, developed by the National Institutes of Health’s Office of Portfolio Analysis. ClinicalTrials.gov provides information on privately and publicly funded clinical studies conducted around the world; iCite, with its modules, measures the influence and translational potential of articles published in PubMed. The MeSH term “proton therapy” was used to search these databases.

## Supplementary Material

MMC1

MMC2

## Figures and Tables

**Table 1:** Summary of randomized clinical trials with results available comparing proton with photon therapy

Reference | ClinicalTrials.gov ID	Title | Status of trial	Objective | No. of participants	Intervention | Radiation type	All-cause mortality | Serious AE | Other AE (%)	Authors’ Comments
Nantavithya et al, 2018^18^ | NCT01511081	SBRT vs SBPT | for high-risk early stage NSCLC; terminated (low accrual)	Phase 2 randomized to compare SBRT vs SBPT for side effects, quality of life, cancer control | 21	SBRT | SBPT	No serious AE with SBRT; metastatic squamous carcinoma of the lung with SBPT^1^ | Other AEs: None	No meaningful comparison could be made between SBRT and SBPT due to low accrual and termination
Laio et al, 2018^19^ | NCT00915005	Bayesian randomized trial of image guided adaptive conformal photon vs PSPT, with concurrent chemotherapy for locally advanced NSCLC: Treatment-related pneumonitis and locoregional recurrence | Completed	Phase 2 randomized trial to study if PSPT compared with IMRT reduces the risk of treatment-related pneumonitis or tumor recurrence in NSCLC | 275	PSPT | Photon therapy | Paclitaxel | Carboplatin	All-cause mortality 2.17% in IMRT group vs 0% in PSPT | PSPT did not improve dosevolume indices for lung but did for heart | Serious AEs were significantly higher in the PSPT group (38.6%) compared with IMRT (30.4%) (*P* < .01) | No difference in other AEs (excluding serious) | Results indicate improvements in endpoints with corresponding improvements in PSPT over the course of the trial	Higher RBE at the distal end of the Bragg peak than used in treatment may have caused higher-thanexpected dose, resulting in higher serious AE with PSPT | Lung is a radiosensitive organ, and any inadvertent exposure with protons is less forgiving due to higher RBE of protons | Organ motion due to breathing caused greater-thanexpected exposure of normal lung volume | Incorporation of predictive biomarkers of normal tissue injury and tumor response is necessary to drive personalization of therapy by dose escalation/deescalation
Lin et al, 2020^26^ | NCT01512589 *	Randomized phase 2B trial of proton beam therapy vs IMRT for locally advanced esophageal cancer	Phase 2 randomized trial to compare proton beam therapy with IMRT in combination with chemotherapy in patients with esophageal cancer | 180	PT | IMRT | Concurrent CRT with fluorouracil and capecitabine with a taxane, or carboplatin with a taxane, or fluorouracil with oxaliplatin | Eligible patients received esophagectomy 810 wk after CRT or after recovery from CRT toxicities	Numerically fewer cardiopulmonary toxicities and postoperative complications in PT arm, which included arterial fibrillation, asymptomatic effusions, lowergrade radiation pneumonitis, ARDS, and reintubation | for patients who completed CRT, there were 3 grade 5 events in the IMRT group | 1 death due to ARDS and 2 due to pulmonary embolism and myocardial infarction	PT provided lower total toxicity burden compared with IMRT but did not improve PFS and QoL | PT may be advantageous in reducing the number and severity of postoperative complications | Results are promising, but a larger multiinstitutional trial is needed for validation of a clear advantage of PT over IMRT for esophageal cancers

*Abbreviations:* AE = adverse event; ARDS = acute respiratory distress syndrome; CRT = chemoradiotherapy; IMRT = intensity modulated radiation therapy; NSCLC = non-small cell lung cancer; PFS = progression-free survival; PSPT = pencil scanning proton therapy; PT = proton therapy; QoL = quality of life; RBE = relative biological effectiveness; RT = radiation therapy; SBPT = stereotactic body proton therapy; SBRT = stereotactic body radiation therapy.

* Results not yet posted at www.clinicaltrials.gov as of December 2020.

**Table 2:** Mean dose in gray (RBE) to critical organs at risk (range) in (NCT00915005)

Organs at risk	IMRT (range)	PSPT (range)	*P*-value
Lung	16.6 (0.4–22.7)	16.1 (6.9–22.1)	.818
Esophagus	23.9 (3.4–47.6)	23.6 (0.0–49.9)	.717
Heart	10.1 (0.6–34.6)	5.9 (0.4–21.1)	.002

*Abbreviations:* IMRT = intensity modulated radiation therapy; PSPT = pencil scanning proton therapy.

Data extracted from Liao et al.^18^

**Table 3:** Summary of issues and future directions in the comparison of proton therapy with photon therapy in the treatment of cancers of various organ/sites

Comparisons
● A head-to-head comparison of PTwith photon therapy could provide meaningful insights for designing future trials with PT. However, the results from a few comparative studies for efficacy and AEs have limitations considering advances in technology, physics, and biology are a continuum and hence needs to be seen as an iterative process in a defined path towards delivering optimal radiation treatment. Nonetheless, for the appropriate clinical use of PT and justification of costs, comparative trials are essential, recognizing that the “ongoing development” is a reality with almost every medical intervention and well-done data at the extant state-of-the-art/ science” are essential for further advancement.
Technology
■ At present, while proton and photon therapies are at different technological maturity levels for engineering, performance, and treatment capacities, the gap is closing to allow/require comparative trials.
Physics
■ Initial comparisons were made between PSPT and photon therapies, which is now evolved to pencil beam scanning PT and IMPT with improved beam delivery, and imaging for target delineation and utilization that has provided better precision and dose distribution.
■ Comparisons of treatment plans or retrospective analysis of data demonstrate dosimetric advantages of PT over photons in the treatment of cancers of many organ sites. Such dosimetric benefit does not ensure PT has more favorable clinical outcomes but does form a basis for comparative prospective trials.
Biology
■ The use of a fixed “average” RBE can simultaneously reduce the advantage of PTand enhance the toxicity at biological “hot spots”. How best to use PT will be an ongoing process that benefits from comparative studies in that there is a mature experience with photons with which to compare PT outcomes.
Clinical observations from the literature review
● Anatomic sites have their normal tissue challenges that limit PT dose. The impact of partial organ treatment that might be possible with PT requires understanding where there may be critical RBE hot spots, the benefit to reduction of integral dose, including a reduction in lymphopenia and immune suppression.
● The ability to precisely target tumors requires an understanding of local tumor biology. For example, brain tumors - due to the infiltrative nature of cancers, both PTand photon therapy limit local control. Irreversible progressive and neurocognitive deficits result in poor quality-of-life among many survivors. Pancreatic tumor treatment is limited by proximity to the small intestine.
● As pilot/single arm interventional studies are ultimately of limited value, conducting large-scale pragmatic prospective RCT is a potential unique approach to comparative PT and photon trials. Such trials:
○ will help confirm or refute the dosimetric advantages of PT over photon therapy
○ should employ better treatment planning incorporating novel biological models that integrate variable RBE and many patientcentric factors (e.g., tumor characteristics, genetics, treatment risks, and responses to treatment), in addition to continuous improvements in beam delivery and on-board imaging for target delineation and utilization.
● It is important that treatment centers are able to keep up with the proven advances in treatment planning and delivery. The complexity and labor intensity of PT required to remain current might lend itself of novel models of centralized sharing of treatment planning.
● The concept of accurate radiation precision medicine should incorporate biomarkers to monitor tumor and normal tissue responses to provide better outcomes to all patients
