# Supplementary material for: Normal Tissue Injury Induced by Photon and Proton Therapies: Gaps and Opportunities
Source: Int J Radiat Oncol Biol Phys. Author manuscript; Available in PMC 2021 Oct 7. (PMC8496269; doi:10.1016/j.ijrobp.2021.02.043)
Supplement: MMC1 [file NIHMS1677927-supplement-MMC1.docx]

**Box 1.** Key issues that contribute to normal tissue injury with proton therapy

| Key Issues | Contributing Factors |
| --- | --- |
| Uncertainties in the depth of penetration of beams | - Differences in tissue compositions - Target movement due to breathing motion, changes in bowel and bladder filling as well as other normal tissue changes |
| RBE: LET, dose, fraction, and tissue type | - Contentious use of a fixed RBE value assigned to protons may overdose normal tissue and underdose tumors - Strong impact of LET on normal tissue injury is not being included in the RBE definition - Not using variable RBE values limits personalization of RT |
| Delineation of the target volume | - Difficulty in achieving adequate tumor delineation due to want of high-quality diagnostic imaging i.e., CT-MRI or PET-MRI |
| Target Motion | - Changes in tumor location during treatment fractions caused by:   - discrepancies between day-to-day patient set up procedures   - breathing   - peristaltic movement   - bowel movement   - heart beats   - organ filling |

**Abbreviations:** CT-MRI, computed tomography-magnetic resonance imaging; LET, linear energy transfer; PET-MRI,

positron emission tomography-magnetic resonance imaging; RBE, relative biological effectiveness; RT, radiotherapy

| Box 2. Summary of issues and future directions in the comparison of proton therapy with photon therapy in the treatment of cancers of various organ/sites |
| --- |
| Comparisons   - A head-to-head comparison of PT with photon therapy could provide meaningful insights for designing future trials with PT. However, the results from a few comparative studies for efficacy and AEs have limitations considering advances in technology, physics, and biology are a continuum and hence needs to be seen as an iterative process in a defined path towards delivering optimal radiation treatment. Nonetheless, for the appropriate clinical use of PT and justification of costs, comparative trials are essential, recognizing that the “ongoing development” is a reality with almost every medical intervention and well-done data at the extant state-of-the-art/science” are essential for further advancement.   Technology   - - - At present, while proton and photon therapies are at different technological maturity levels for engineering, performance, and treatment capacities, the gap is closing to allow/require comparative trials.   Physics   - - - Initial comparisons were made between PSPT and photon therapies, which is now evolved to pencil beam scanning PT and IMPT with improved beam delivery, and imaging for target delineation and utilization that has provided better precision and dose distribution.     - Comparisons of treatment plans or retrospective analysis of data demonstrate dosimetric advantages of PT over photons in the treatment of cancers of many organ sites. Such dosimetric benefit does not ensure PT has more favorable clinical outcomes but does form a basis for comparative prospective trials.   Biology   - - - The use of a fixed “average” RBE can simultaneously reduce the advantage of PT and enhance the toxicity at biological “hot spots”. How best to use PT will be an ongoing process that benefits from comparative studies in that there is a mature experience with photons with which to compare PT outcomes.   Some clinical observations from the literature review include the following.   - Anatomic sites have their normal tissue challenges that limit PT dose. The impact of partial organ treatment that might be possible with PT requires understanding where there may be critical RBE hot spots, the benefit to reduction of integral dose, including a reduction in lymphopenia and immune suppression. - The ability to precisely target tumors requires an understanding of local tumor biology. For example, brain tumors - due to the infiltrative nature of cancers, both PT and photon therapy limit local control. Irreversible progressive and neurocognitive deficits result in poor quality-of-life among many survivors. Pancreatic tumor treatment is limited by proximity to the small intestine. - As pilot/single arm interventional studies are ultimately of limited value, conducting large-scale pragmatic prospective RCT is a potential unique approach to comparative PT and photon trials. Such trials:   - will help confirm or refute the dosimetric advantages of PT over photon therapy   - should employ better treatment planning incorporating novel biological models that integrate variable RBE and many patient-centric factors (*e.g.*, tumor characteristics, genetics, treatment risks, and responses to treatment), in addition to continuous improvements in beam delivery and on-board imaging for target delineation and utilization.   - It is important that treatment centers are able to keep up with the proven advances in treatment planning and delivery. The complexity and labor intensity of PT required to remain current might lend itself of novel models of centralized sharing of treatment planning.   - The concept of accurate radiation precision medicine should incorporate biomarkers to monitor tumor and normal tissue responses to provide better outcomes to all patients. |

**Abbreviations:** AE, Adverse events; IMPT, Intensity modulated proton therapy; PSPT, Passive scattering proton therapy; PT, proton therapy; RBE, Relative biological effectiveness; RCT, randomized clinical trial; RT, radiotherapy
