# Supplementary figures and images for: Normal Tissue Injury Induced by Photon and Proton Therapies: Gaps and Opportunities
Source: Int J Radiat Oncol Biol Phys. Author manuscript; Available in PMC 2021 Oct 7. (PMC8496269; doi:10.1016/j.ijrobp.2021.02.043)

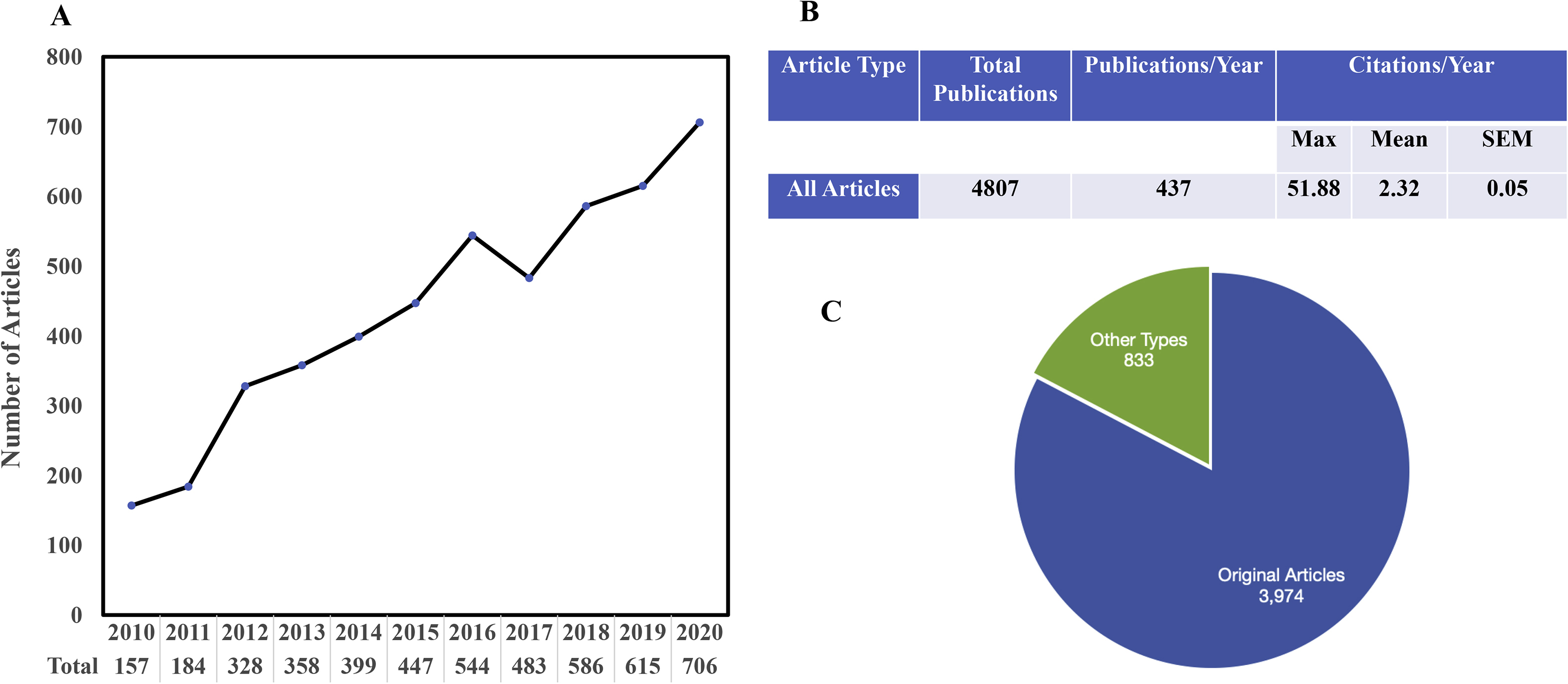

Supplement: MMC2 [file NIHMS1677927-supplement-MMC2.jpg]
